# Structure of the HIV-1 reverse transcriptase Q151M mutant: insights into the inhibitor resistance of HIV-1 reverse transcriptase and the structure of the nucleotide-binding pocket of *Hepatitis B virus* polymerase

**DOI:** 10.1107/S2053230X15017896

**Published:** 2015-10-23

**Authors:** Akiyoshi Nakamura, Noriko Tamura, Yoshiaki Yasutake

**Affiliations:** a Bioproduction Research Institute, National Institute of Advanced Industrial Science and Technology (AIST), 2-17-2-1 Tsukisamu-Higashi, Toyohira, Sapporo, Hokkaido 062-8517, Japan

**Keywords:** crystal structure, viral protein, reverse transcriptase, *Hepatitis B virus*

## Abstract

*Hepatitis B virus* polymerase (HBV Pol) is an important target for anti-HBV drug development; however, its low solubility and stability **in vitro** has hindered detailed structural studies. Certain nucleotide reverse transcriptase (RT) inhibitors (NRTIs) such as tenofovir and lamivudine can inhibit both HBV Pol and *Human immunodeficiency virus 1* (HIV-1) RT, leading to speculation on structural and mechanistic analogies between the deoxynucleotide triphosphate (dNTP)-binding sites of these enzymes. The Q151M mutation in HIV-1 RT, located at the dNTP-binding site, confers resistance to various NRTIs, while maintaining sensitivity to tenofovir and lamivudine. The residue corresponding to Gln151 is strictly conserved as a methionine in HBV Pol. Therefore, the structure of the dNTP-binding pocket of the HIV-1 RT Q151M mutant may reflect that of HBV Pol. Here, the crystal structure of HIV-1 RT Q151M, determined at 2.6 Å resolution, in a new crystal form with space group *P*321 is presented. Although the structure of HIV-1 RT Q151M superimposes well onto that of HIV-1 RT in a closed conformation, a slight movement of the β-strands (β2–β3) that partially create the dNTP-binding pocket was observed. This movement might be caused by the introduction of the bulky thioether group of Met151. The structure also highlighted the possibility that the hydrogen-bonding network among amino acids and NRTIs is rearranged by the Q151M mutation, leading to a difference in the affinity of NRTIs for HIV-1 RT and HBV Pol.

## Abstract 2

The structure of the HIV-1 reverse transcriptase Q151M mutant was determined at a resolution of 2.6 Å in space group *P*321.

## 1. Introduction   

*Hepatitis B virus* (HBV) infection is a global health problem, with approximately 400 million chronically infected patients worldwide (Lavanchy, 2004 ▸). The infection causes serious liver diseases, resulting in one million deaths per year (Ott *et al.*, 2012 ▸). HBV contains a partially double-stranded DNA genome (∼3.2 kb) that is replicated by reverse transcription of pregenomic RNA in a viral nucleocapsid particle (Wang *et al.*, 2014 ▸; Seeger & Mason, 2000 ▸; Summers & Mason, 1982 ▸). The reverse transcription is catalyzed by HBV polymerase (Pol), comprising four functionally distinct domains: a terminal protein (TP) domain, a spacer region, a reverse transcriptase (RT) domain and a C-terminal ribonuclease H (RH) domain (Radziwill *et al.*, 1990 ▸; Toh *et al.*, 1983 ▸). The RT and RH domains exhibit amino-acid sequence homology to those of retroviral RTs. Since HBV Pol is vital to the viral life cycle, several nucleoside analogues that inhibit the RT activity of HBV Pol have been developed (Michailidis *et al.*, 2012 ▸; Zoulim, 2011 ▸). Currently, all approved chemotherapeutics for HBV are nucleotide RT inhibitors (NRTIs; Nassal, 2009 ▸). Initially, NRTIs show efficient inhibition of HBV growth; however, with long-term treatment they tend to select for drug-resistant HBV strains (Hayashi *et al.*, 2015 ▸; Mukaide *et al.*, 2010 ▸; Zoulim, 2011 ▸; Matthews, 2006 ▸). Therefore, novel RT inhibitors are necessary for the development of combination therapies against chronic HBV infections. Although large-scale purification of recombinant HBV Pol by refolding has recently been reported (Voros *et al.*, 2014 ▸), it is still a challenging process to obtain active and stable HBV Pol **in vitro** for X-ray structural analyses and high-throughput drug screening.

Although some NRTIs (for example tenofovir and lamivudine) were developed for the treatment of *Human immunodeficiency virus 1* (HIV-1) infection, their potency against HBV infection has been recognized, suggesting that the NRTI (dNTP)-binding sites of both RTs share a similar structure. Therefore, homology models based on the HIV-1 RT structure have been used in previous studies to explain putative HBV Pol–NRTI interactions (Das *et al.*, 2001 ▸; Langley *et al.*, 2007 ▸; Mukaide *et al.*, 2010 ▸). With regard to HIV-1 RT dNTP-binding residues, the only difference between HIV-1 RT and HBV Pol is Gln151: the corresponding residue in HBV Pol is absolutely conserved as a methionine (Poch *et al.*, 1989 ▸; Wang *et al.*, 2012 ▸). It has been reported that a Q151M mutation in HIV-1 RT induces the development of NRTI resistance in mutant viruses (Shirasaka *et al.*, 1993 ▸, 1995 ▸). It is also known that the Q151M mutant confers resistance to almost all NRTIs, while maintaining sensitivity to lamivudine and tenofovir (Iversen *et al.*, 1996 ▸; Mbisa *et al.*, 2011 ▸; Harada *et al.*, 2007 ▸). Consistent with these results, among anti-HIV-1 NRTIs only lamivudine and tenofovir have been approved for treatment of HBV infection. Moreover, the Q151M mutant appears to confer hypersensitivity to entecavir, a powerful NRTI used for the treatment of HBV infection (Zennou *et al.*, 2007 ▸). Considering these aspects, we performed a crystallographic study of the HIV-1 RT Q151M mutant to investigate the mutational effect on HIV-1 RT and the structural details of the HBV Pol dNTP-binding site.

## 2. Materials and methods   

### 2.1. Protein expression and purification   

The genes encoding HIV-1 RT p66 and p51 used in this study originated from the HIV-1 clone pNL4-3 (GenBank M19921.2). The p51 gene fragment was inserted into the NdeI/XhoI sites of a modified pET-28b vector (Novagen), in which a His_6_ tag was fused at the N-terminus (pET-28_His_6_-p51). The p66 gene fragment was cloned into the NcoI/XhoI sites of a pCDF-Duet vector (Novagen). The Q151M mutation was introduced into the p66 gene of the pCDF-Duet vector (pCDF_p66_Q151M) by inverse PCR according to a previously described method (Hemsley *et al.*, 1989 ▸). *Escherichia coli* BL21-CodonPlus (DE3)-RIL strain (Novagen) was co-transformed with pET-28_His_6_-p51 and pCDF_p66_Q151M by electroporation. Cells were grown at 37°C in LB medium containing 20 µg ml^−1^ kanamycin and 25 µg ml^−1^ spectinomycin. Expression of HIV-1 RT Q151M p66/p51 was induced by adding 0.1 m*M* isopropyl β-d-1-thiogalactopyranoside (IPTG) for a further 16 h at 25°C. The cells were harvested by centrifugation (6693*g*, 15 min, 4°C). The resultant cell pellet was then suspended in buffer *A* (50 m*M* sodium phosphate pH 8.0, 300 m*M* NaCl, 2 m*M* MgCl_2_, 10% glycerol) and disrupted using a sonicator (TOMY) in buffer *A* with 1 mg ml^−1^ lysozyme and 25 U ml^−1^ Benzonase (Merck). Cell debris was removed by centrifugation (27 216*g*, 20 min, 4°C). The supernatant was loaded onto an Ni-affinity column (Sigma–Aldrich) pre-equilibrated with buffer *A*. The column was washed with buffer *A* followed by buffer *B* (50 m*M* sodium phosphate pH 6.0, 300 m*M* NaCl, 2 m*M* MgCl_2_, 10% glycerol), and the bound proteins were eluted with a linear gradient of 0–400 m*M* imidazole in buffer *B*. The pooled fractions were dialyzed against buffer *C* [50 m*M* Tris–HCl pH 8.0, 2 m*M* MgCl_2_, 1 m*M* dithiothreitol (DTT), 10% glycerol] and then loaded onto a DEAE Sepharose Fast Flow column (GE Healthcare). The sample was collected in a flowthrough fraction and its purity was examined by SDS–PAGE. The purified HIV-1 RT Q151M p66/p51 was dialyzed against buffer *D* (20 m*M* Tris–HCl pH 8.0, 2 m*M* MgCl_2_, 1 m*M* DTT) and was concentrated to 8 mg ml^−1^ using a centrifugal filtration device (50 kDa molecular-weight cutoff; Millipore). The protein concentration was determined by the Bradford protein assay (Bio-Rad) using bovine serum albumin as a standard.

### 2.2. Crystallization   

Initial crystallization screening was performed using Crystal Screen, Crystal Screen 2, Index, PEGRx, PEG/Ion (Hampton Research), The PACT Suite (Qiagen) and Wizard I and II (Emerald Bio) by the sitting-drop vapour-diffusion method in 96-well plates. Drops were comprised of 0.1 µl sample and an equal volume of reservoir solution and were equilibrated against 70 µl reservoir solution at 20°C. Subsequent optimization of the initial hit conditions was performed using hanging-drop vapour diffusion at 20°C by changing the pH value of the reservoir solution and the concentrations of the buffer and precipitant, and by using various additives. The hanging drops were set up by mixing 1.5 µl sample solution with 1.5 µl reservoir solution and were equilibrated against 500 µl reservoir solution in 24-well plates. The initial screening yielded crystals in two conditions: (i) 0.1 *M* bicine pH 8.5, 15%(*w*/*v*) PEG 1500 (PEGRx condition No. 21) and (ii) 0.1 *M* imidazole pH 8.0, 10%(*w*/*v*) PEG 8000 (Wizard II condition No. 34). The small and fragile crystals obtained from the former condition could not be improved, while single crystals could be obtained by optimizing the latter condition. Finally, well diffracting crystals (0.2 × 0.2 × 0.2 mm) were produced with reservoir solution consisting of 0.2 *M* imidazole pH 8.0, 9%(*w*/*v*) PEG 8000.

### 2.3. Data collection, structure determination and refinement   

Prior to data collection, the crystal was transferred stepwise into a cryoprotectant solution consisting of 0.2 *M* imidazole pH 8.0, 12%(*w*/*v*) PEG 8000 with increasing concentrations of ethylene glycol up to 25%(*v*/*v*), and was flash-cooled in a liquid-nitrogen gas stream at 100 K. An X-ray diffraction data set for HIV-1 RT Q151M was collected using a PILATUS3 6M detector (Dectris) on beamline BL-17A at the Photon Factory, Tsukuba, Japan at a radiation wavelength of 0.98000 Å. The raw image data were processed using the *HKL*-2000 package (Otwinowski & Minor, 1997 ▸). The HIV-1 RT Q151M crystals belonged to space group *P*321, with unit-cell parameters *a* = *b* = 145.74, *c* = 118.40 Å.

The structure of HIV-1 RT Q151M was solved by molecular replacement using *MOLREP* (Vagin & Teplyakov, 2010 ▸) in the *CCP*4 suite (Winn *et al.*, 2011 ▸). The molecular-replacement solutions obtained revealed one p66/p51 heterodimer of HIV-1 RT Q151M in the asymmetric unit. The atomic model was rebuilt and modified manually using *Coot* (Emsley *et al.*, 2010 ▸). Model refinement was performed using *REFMAC*5 (Murshudov *et al.*, 2011 ▸) and *phenix.refine* (Afonine *et al.*, 2012 ▸). Finally, the *R*_work_ and *R*_free_ factors converged to 20.3 and 23.9%, respectively. Data-collection and refinement statistics are presented in Table 1 ▸. *MolProbity* (Chen *et al.*, 2010 ▸) was used for model validation. All figures were generated using *PyMOL* (Schrödinger). The atomic coordinates and structure factors have been deposited in the RCSB Protein Data Bank under accession code 4zhr.

## 3. Results and discussion   

### 3.1. Overall structure description   

**Figure 1:**
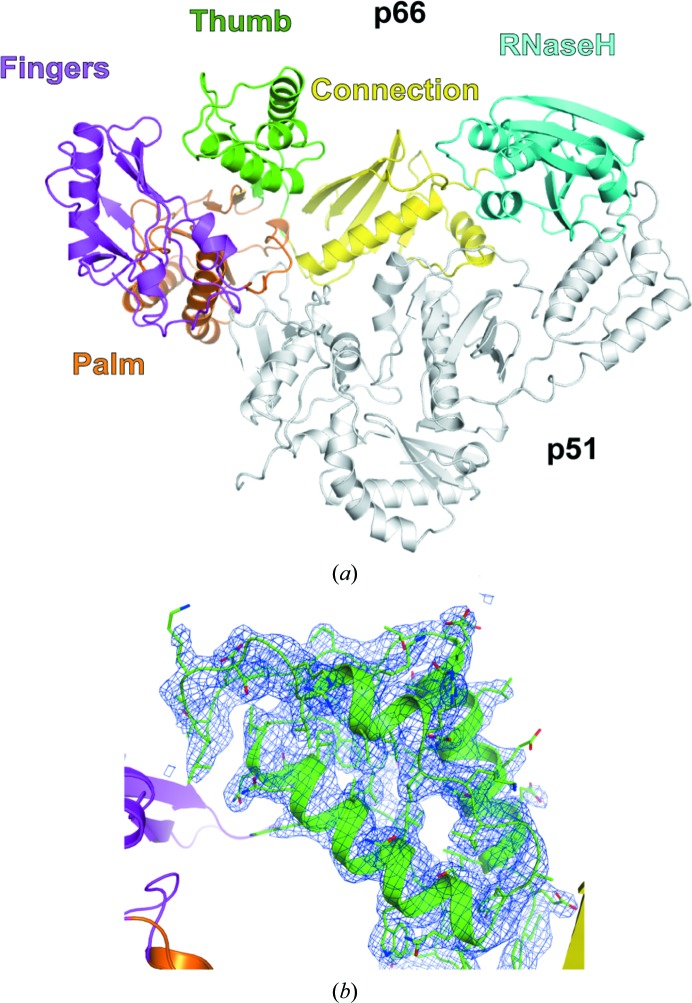
Overall structure of the HIV-1 RT Q151M mutant. (*a*) Ribbon diagram of the HIV-1 RT Q151M mutant. The fingers, palm, thumb, connection and RH domains/subdomains of the p66 subunit are shown in magenta, orange, green, yellow and cyan, respectively. The p51 subunit is shown in grey. (*b*) The 2*mF*_o_ − *DF*_c_ map of the thumb subdomain is shown in blue contoured at the 1.0σ level.

The crystal structure of HIV-1 RT Q151M was determined at 2.6 Å resolution (Fig. 1 ▸ *a*). HIV-1 RT Q151M was crystallized in the trigonal space group *P*321, with one p66/p51 heterodimer in the asymmetric unit. The structure was determined by molecular replacement using an inhibitor-bound HIV-1 RT structure (PDB entry 1rth; Ren *et al.*, 1995 ▸) as a search model. The electron-density map after molecular replacement showed significant movement of the thumb subdomain as described below; therefore, the model of the thumb subdomain was deleted completely once and then rebuilt manually. The refined HIV-1 RT Q151M structure is well ordered, and the maps show clear electron density for the rebuilt thumb subdomain (Fig. 1 ▸ *b*). Owing to poor electron density, the residues Glu89–Gly93 and Asp218–Met230 of the p51 subunit were not modelled. p66 is a catalytic subunit that consists of two functional domains: an N-terminal RT domain (residues 1–426) and a C-terminal RH domain (residues 427–560) (Sarafianos *et al.*, 2009 ▸). The N-terminal RT domain is further divided into four subdomains: the fingers (residues 1–85 and 118–155), the palm (residues 86–117 and 156–237), the thumb (residues 238–318) and the connection (residues 319–426) subdomains (Fig. 1 ▸ *a*).

**Figure 2:**
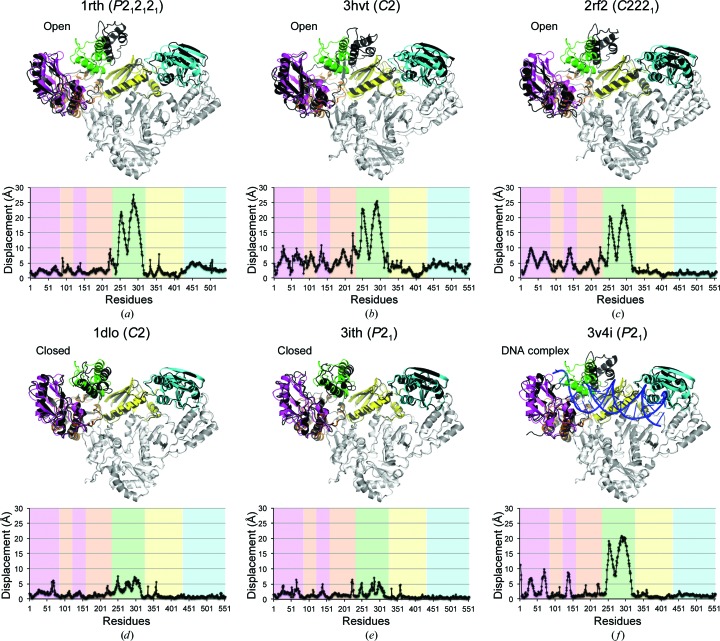
Comparison of HIV-1 RT structures crystallized in different space groups. HIV-1 RT structures were aligned by the residues of p51 using *Chimera* (Pettersen *et al.*, 2004 ▸). The displacement values of the residues of the p66 subunit between HIV-1 RT Q151M and six HIV-1 RT representatives were calculated using *Chimera*. The colours used to indicate HIV-1 RT Q151M are the same as those used in Fig. 1 ▸. Other HIV-1 RT structures are coloured black (p66) and grey (p51). The colours in the graphs correspond to the colours of p66. (*a*) PDB entry 1rth, *P*2_1_2_1_2_1_; (*b*) PDB entry 3hvt, *C*2; (*c*) PDB entry 2rf2, *C*222_1_; (*d*) PDB entry 1dlo, *C*2; (*e*) PDB entry 3ith, *P*2_1_; (*f*) PDB entry 3v4i, bound to DNA, *P*2_1_.

A large number of crystal structures of HIV-1 RT as the apoenzyme (Hsiou *et al.*, 1996 ▸), inhibitor-bound forms (Sarafianos *et al.*, 2002 ▸; Tuske *et al.*, 2004 ▸; Lindberg *et al.*, 2002 ▸; Wang *et al.*, 1994 ▸) and oligonucleotide-bound forms (Huang *et al.*, 1998 ▸; Jacobo-Molina *et al.*, 1993 ▸; Peletskaya *et al.*, 2004 ▸; Sarafianos *et al.*, 2001 ▸; Lapkouski *et al.*, 2013 ▸) have been reported. The previously reported apo and inhibitor-bound HIV-1 RT crystals are classified into four different space groups: *P*2_1_2_1_2_1_ (representative PDB entry 1rth; Ren *et al.*, 1995 ▸), *C*2 [PDB entries 3hvt (Smerdon *et al.*, 1994 ▸) and 1dlo (Hsiou *et al.*, 1996 ▸)], *C*222_1_ (PDB entry 2rf2; Zhao *et al.*, 2008 ▸) and *P*2_1_ (PDB entry 3ith; Freisz *et al.*, 2010 ▸). The p51 subunit of HIV-1 RT Q151M superposed very well onto each of the HIV-1 RT structures, *i.e.* PDB entries 1rth (r.m.s.d. of 0.954 Å for 364 C^α^ atoms), 3htv (r.m.s.d. of 1.123 Å for 256 C^α^ atoms), 2rf2 (r.m.s.d. of 0.971 Å for 370 C^α^ atoms), 1dlo (r.m.s.d. of 0.598 Å for 371 C^α^ atoms), 3ith (r.m.s.d. of 0.618 Å for 373 C^α^ atoms) and 3v4i (r.m.s.d. of 0.651 Å for 376 C^α^ atoms), indicating the rigidity of the p51 subunit, which functions as a structural scaffold. In contrast, the relative orientations of the RT subdomains of the p66 subunit differ substantially (Fig. 2 ▸). It is known that the thumb subdomain can exhibit ‘open’ and ‘closed’ conformations in the DNA-bound or NRTI-bound HIV-1 RT structures and unliganded structures, respectively (Hsiou *et al.*, 1996 ▸; Wright *et al.*, 2012 ▸). The displacement of each residue between p66 of HIV-1 RT Q151M and that of other HIV-1 RTs in the open conformation (PDB entries 1rth, 3htv and 2rf2) showed a large movement of the thumb subdomain (∼25 Å) and a slight movement of the other subdomains (∼5 Å) (Figs. 2 ▸ *a*, 2 ▸ *b* and 2 ▸ *c*). In contrast, the relative orientations of the p66 subdomains of HIV-1 RT Q151M superposed well onto those of the closed structures (PDB entries 1dlo and 3ith; Figs. 2 ▸ *d* and 2 ▸ *e*). These structural comparisons among various types of HIV-1 RTs revealed that HIV-1 RT Q151M forms the closed conformation. The structural comparisons also showed that the thumb subdomain and the β2–β3 strands (residue 60–75) in the finger subdomain are relatively mobile in any conformational state (Fig. 2 ▸). It has been revealed that the p66 thumb subdomain partially binds to the minor groove of the DNA and that the β2–β3 strands are located between the ends of the primer and template strands in the HIV-1 RT–DNA complex structure (Jacobo-Molina *et al.*, 1993 ▸). Considering these observations, the mobilities of the thumb subdomain and the β2–β3 strands are likely to be involved in recognition of the template oligo­nucleotide and the substrate dNTP. The thumb subdomain and β2–β3 strands also vary slightly among the closed structures (∼5 Å; Figs. 2 ▸ *d* and 2 ▸ *e*). Of these two regions, the conformation of the β2–β3 strands in the HIV-1 RT Q151M structure is different from those in any of the other HIV-1 RT structures in the closed conformation (PDB entries 1dlo, 3ith and 1hmv; Rodgers *et al.*, 1995 ▸), which might be caused by the Q151M mutation, as described below.

### 3.2. Structural comparison of the dNTP-binding pocket between NRTI-bound HIV-1 RT and the Q151M mutant   

**Figure 3:**
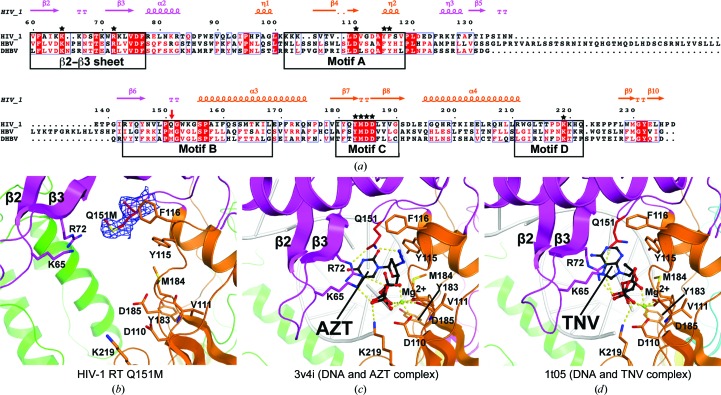
Comparison of the dNTP-binding sites. (*a*) Multiple sequence alignment of HIV-1 RT, human HBV Pol RT domain and *Duck hepatitis B virus* (DHBV) Pol RT domain. Secondary structures of HIV-1 RT are indicated at the top (α, α-helix; β, β-strand; η, 3_10_-helix). The Q151M mutational point is indicated by a red arrow. Residues forming the dNTP-binding site are denoted by black stars. The conserved motifs among the various types of RTs are indicated by black boxes. Each protein sequence was aligned by *ClustalW* (Larkin *et al.*, 2007 ▸) and the figure was prepared with *ESPript* (Robert & Gouet, 2014 ▸). (*b*), (*c*) and (*d*) show the dNTP-binding pockets of HIV-1 RT Q151M, HIV-1 RT with bound DNA and azidothymidine triphosphate (AZT; PDB entry 3v4i) and HIV-1 RT with bound DNA and tenofovir diphosphate (TNV; PDB entry 1t05), respectively. Residues that form the dNTP-binding site are displayed as stick models. The final 2*mF*_o_ − *DF*_c_ map of Met151 is shown in blue contoured at the 1.0σ level. DNA molecules are shown as white ribbon models. AZT and TNV are coloured black and shown as ball-and-stick models. Green spheres indicate Mg^2+^ ions coordinated to the phosphate groups of the inhibitors and Asp110 and Asp185. Dashed lines indicate hydrogen bonds in the dNTP-binding pocket.

The RT domain of human HBV Pol exhibits weak amino-acid sequence homology (∼20%) to HIV-1 RT (Fig. 3 ▸ *a*). It has been proposed that the short motifs, boxes A–E (Fig. 3 ▸ *a*) and boxes F and G, are universally conserved (Wang *et al.*, 2012 ▸). Since certain NRTIs such as lamivudine and tenofovir are effective against both HBV and HIV-1 RTs, it seems likely that the structure of the dNTP-binding pocket is similar in both RTs. HIV-1 RT with a Q151M mutation is known to exhibit NRTI resistance (Shirasaka *et al.*, 1993 ▸, 1995 ▸), while it remains sensitive to tenofovir and lamivudine (Iversen *et al.*, 1996 ▸; Mbisa *et al.*, 2011 ▸). It should be noted that the methionine residue corresponding to Gln151 in HIV-1 RT is absolutely conserved in the HBV RT domain (Poch *et al.*, 1989 ▸). Additionally, the methionine is the only residue within the dNTP-binding pocket that is not conserved between the HIV-1 and HBV RTs. Therefore, we propose that the structure of the dNTP-binding pocket of HIV-1 RT Q151M may provide some clues regarding the dNTP/NRTI-binding pocket of HBV Pol.

The dNTP-binding site of HIV-1 RT Q151M was compared with azidothymidine triphosphate (AZT)-bound (PDB entry 3v4i; Das *et al.*, 2012 ▸) and tenofovir diphosphate (TNV)-bound (PDB entry 1t05; Tuske *et al.*, 2004 ▸) HIV-1 RT structures (Fig. 3 ▸). The dNTP-binding site is composed of three aspartate residues in motifs A (Asp110) and C (Asp185 and Asp186) in the p66 palm subdomain that bind Mg^2+^ ions required for catalysis (Larder *et al.*, 1987 ▸; Sarafianos *et al.*, 2009 ▸). Moreover, two positively charged residues, Lys65 and Arg72, are involved in binding to the β- and γ-phosphate groups of the incoming dNTP (Huang *et al.*, 1998 ▸), AZT (Das *et al.*, 2012 ▸) (Fig. 3 ▸ *c*) and TNV (Tuske *et al.*, 2004 ▸; Fig. 3 ▸ *d*). Tyr115 in motif A recognizes the deoxyribose ring of the incoming dNTP and discriminates between dNTP and NTP (Boyer *et al.*, 2000 ▸). Gln151 in motif B interacts directly with the 3′-OH of the incoming dNTP and the conserved Arg72 *via* hydrogen bonds (Huang *et al.*, 1998 ▸). Gln151 in the AZT-bound structure also forms a hydrogen bond to the azide group of AZT (Fig. 3 ▸ *c*), whereas this is not the case in the TNV-bound structure. Instead, the small hydrophobic group (methyl group) of TNV is located close to the Gln151 side chain (Fig. 3 ▸ *d*). It is evident that the Q151M mutation causes a loss of the capacity to form hydrogen bonds; therefore, the abovementioned hydrogen bonds could not be formed in the structure of the Q151M mutant. The structural analysis in this study also showed clear electron density for the Met151 residue, which indicated that the side chain of Met151 is flipped out from the dNTP-binding pocket and is exposed to the solvent. In addition, the bulky thioether group of Met151 further promoted the relocation of the Lys65 and Arg72 side chains and mobile β2–β3 strands involved in the recognition of the β- and γ-phosphate groups of dNTP/NRTI (Fig. 3 ▸ *b*, 3 ▸ *c* and 3 ▸ *d*). Therefore, it is possible that the hydrogen-bonding network between amino acids and inhibitors cannot be formed in the structure of the Q151M mutant (Fig. 3 ▸c and 3 ▸ *d*), thereby leading to alterations in the binding affinities for NRTIs. The structural comparison also suggests that hydrophobic interactions between the methyl group of TNV and the thioether group of methionine might compensate for the loss of hydrogen bonds; hence, tenofovir exhibits an inhibitory action towards both HIV-1 RT Q151M and HBV Pol. There could be a similar binding mechanism for entecavir, involving a hydrophobic exocyclic methylene-group moiety.

The HIV-1 RT Q151M structure reported in this study may serve as the basis for an atomic model for molecular-dynamics and/or docking-simulation studies. Future attempts to accumulate HBV Pol-type mutations for residues in the vicinity of the HIV-1 RT active site would further contribute to our understanding of the mechanisms associated with the different binding affinities of NRTIs for HIV-1 RT and HBV Pol.

## Supplementary Material

PDB reference: HIV-1 reverse transcriptase Q151M mutant, 4zhr

## Figures and Tables

**Table 1:** Data-collection and refinement statistics for HIV-1 RT Q151M Values in parentheses are for the outermost resolution shell.

PDB code	4zhr
Data collection	
Beamline	BL-17A, Photon Factory
Wavelength ()	0.98000
Temperature (K)	100
Detector	PILATUS3 6M
Space group	*P*321
Unit-cell parameters ()	*a* = *b* = 145.74, *c* = 118.40
Resolution ()	502.60 (2.642.60)
Total reflections	1166372
Unique reflections	44912
*R*_merge_ †	0.067 (0.79)
Mean *I*/(*I*)	19.2 (2.1)
Completeness (%)	100.0 (100.0)
Multiplicity	10.0 (9.4)
Refinement	
No. of reflections	44866
*R*_work_ ‡/*R*_free_ §	0.203/0.239
No. of atoms	
Total	7909
Water	38
*B* factors (^2^)	
Overall	72.0
Water	62.2
R.m.s.d. from ideal	
Bond lengths ()	0.002
Bond angles ()	0.54
Ramachandran plot¶	
Favoured (%)	97.38
Allowed (%)	2.62
Outliers (%)	0.00

† *R*_merge_ = 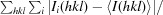 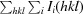, where *I*(*hkl*) is the mean intensity of a set of equivalent reflections.

‡ *R*_work_ = 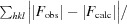 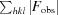 for 95% of the reflection data used in refinement. *F*_obs_ and *F*_calc_ are the observed and calculated structure-factor amplitudes, respectively.

§ *R*_free_ is the equivalent of *R*_work_ except that it was calculated for a randomly chosen 5% test set excluded from refinement.

¶ Ramachandran analysis was performed using *MolProbity* (Chen *et al.*, 2010 ▸).
